# Reef fish communities are spooked by scuba surveys and may take hours to recover

**DOI:** 10.7717/peerj.4886

**Published:** 2018-05-24

**Authors:** Michael J. Emslie, Alistair J. Cheal, M. Aaron MacNeil, Ian R. Miller, Hugh P.A. Sweatman

**Affiliations:** 1 Australian Institute of Marine Science, Townsville, QLD, Australia; 2 Department of Biology, Dalhousie University, Halifax, NS, Canada

**Keywords:** Reef fishes, Diver disturbance, Underwater visual surveys, Monitoring

## Abstract

Ecological monitoring programs typically aim to detect changes in the abundance of species of conservation concern or which reflect system status. Coral reef fish assemblages are functionally important for reef health and these are most commonly monitored using underwater visual surveys (UVS) by divers. In addition to estimating numbers, most programs also collect estimates of fish lengths to allow calculation of biomass, an important determinant of a fish’s functional impact. However, diver surveys may be biased because fishes may either avoid or are attracted to divers and the process of estimating fish length could result in fish counts that differ from those made without length estimations. Here we investigated whether (1) general diver disturbance and (2) the additional task of estimating fish lengths affected estimates of reef fish abundance and species richness during UVS, and for how long. Initial estimates of abundance and species richness were significantly higher than those made on the same section of reef after diver disturbance. However, there was no evidence that estimating fish lengths at the same time as abundance resulted in counts different from those made when estimating abundance alone. Similarly, there was little consistent bias among observers. Estimates of the time for fish taxa that avoided divers after initial contact to return to initial levels of abundance varied from three to 17 h, with one group of exploited fishes showing initial attraction to divers that declined over the study period. Our finding that many reef fishes may disperse for such long periods after initial contact with divers suggests that monitoring programs should take great care to minimise diver disturbance prior to surveys.

## Introduction

Estimating the density of animals is a fundamental part of ecological and monitoring studies. In marine systems and coral reefs in particular, the density of reef fishes has historically been estimated using both destructive and non-destructive sampling techniques. Destructive sampling, such as ichthyocides (e.g. rotenone Randall, 1963; Brock, 1982; Bellwood et al., 2006) or dynamite (Williams & Hatcher, 1983) have advantages in that they provide non-selective estimates of the density and size structure of reef fishes, including highly cryptic species that are often overlooked using other techniques. The obvious disadvantage of destructive techniques is the removal of fishes, corals and other reef invertebrates that are the subject of monitoring.

The use of non-destructive techniques such as underwater visual surveys (UVS), sometimes referred to as underwater visual census, was first proposed by Brock (1954) and has since become one of the most common techniques for estimating reef fish numbers. UVS are relatively inexpensive and, if observers are adequately trained, they are technically simple compared with destructive sampling, providing quick estimates of diversity, abundance and length frequency distributions. There are a number of variants of UVS, the most popular are based either on fixed points or transects. Point counts involve a single observer enumerating all fishes within a cylinder of pre-determined diameter and height for a set period of time (Bohnsack & Bannerot, 1986). Transect surveys involve one or more observers swimming for a defined distance or a set time and counting all individuals within a strip of pre-determined width using instantaneous or non-instantaneous counts (Williams, 1982; Russ, 1984a, 1984b; Bouchon-Navaro & Harmelin-Vivien, 1981). Variations on transect based approaches include estimating the distance to individual fishes or schools on variable width transects to include detectability (Kulbicki, 1998; Kulbicki & Sarramégna, 1999). Recently, the use of video cameras for fish surveys has increased, used singly or as a stereo-pair, baited, un-baited or diver-operated. The use of video has its own difficulties and biases, such as problems identifying distant individuals to species, and difficulty estimating lengths of individuals that are only partially in view (Wilson et al., 2018). There is also considerable handling time to extract information from videos.

Numerous studies have compared the accuracy, efficiency and biases of different methods (Brock, 1982; Sale & Sharp, 1983; Thresher & Gunn, 1986; Cheal & Thompson, 1997; Thompson & Mapstone, 1997; Samoilys & Carlos, 2000; Colvocoresses & Acosta, 2007; Colton & Swearer, 2010). For example, estimates of reef fish density from transect based UVS will be influenced by the level of experience of the observer (correct identification and accurate length estimation), their swimming speed, ability to exclude individuals that have already been counted (Watson, Carlos & Samoilys, 1995), accuracy of estimates of transect width (Sale & Sharp, 1983; Harmelin-Vivien et al., 1985; Fowler, 1987; McCormick & Choat, 1987; Cheal & Thompson, 1997), behaviour of target fishes towards a diver (attraction or evasion) (Kulbicki, 1998) and detectability of target species (Kulbicki, 1998; MacNeil et al., 2008; Feary et al., 2011; Bozec et al., 2011). All of these biases can affect the accuracy and precision of the estimates by different observers for diverse species at different sites and times (Edgar, Barrett & Morton, 2004), and will contribute to sampling error. Biases that remain constant over different sites, times and observers rarely affect the results and interpretation of studies, while those that vary in space and time can result in misleading interpretations (Edgar, Barrett & Morton, 2004).

The presence of SCUBA divers often influences the behaviour of the fishes they wish to count: some fishes are attracted to divers, while others flee with varying flight initiation distances (Ydenberg & Dill, 1986; Januchowski-Hartley et al., 2011; Lindfield et al., 2014). Such behaviours can have significant effects on estimates of reef fish density (Chapman et al., 1974; Kulbicki, 1998; Kulbicki & Sarramégna, 1999). When using point counts, the effect of divers can be controlled by allowing the fishes time to adjust to presence of a diver before counting begins (Bohnsack & Bannerot, 1986). No adjustment period is possible in surveys that use instantaneous strip transects, as the diver is continuously moving. The effects of divers have been estimated using counts of fishes from static video cameras, with and without the presence of divers (Dearden, Theberge & Yasué, 2010) or by counting fishes on a sequence of passes of the same transect (Dickens et al., 2011). The number of individuals recorded declined significantly in the presence of divers, while the extent of decline varied among taxa. While many studies have documented declines in fish abundance due to the presence of divers, few have tried to estimate the time taken for reef fishes to return to pre-disturbance levels after disturbance by divers. Somerton, Williams & Campbell (2017) examined this question qualitatively using remote cameras for the Caribbean Vermillion snapper (*Rhomboplites aurorubens*, Lutjanidae) and estimated that abundance returned to pre-disturbance levels after about 20 min. Additionally, Dearden, Theberge & Yasué (2010) found no difference in fish numbers seen on video before and after 10 min UVS counts by divers, despite a reduction in fish abundance recorded by videos during the surveys. They concluded that fishes returned rapidly once divers had left the area. However, as neither of these studies used belt transects, the return time of reef fishes following disturbance by divers conducting UVS by continuously moving down belt transects remains unknown.

Underwater visual surveys are frequently used to document differences in reef fish assemblages between no-take marine reserves and areas that are open to fishing, or to compare temporal dynamics of reef fish populations across different spatial scales. The long-term monitoring program (LTMP) at the Australian Institute of Marine Science (AIMS) quantifies the dynamics of coral reef fishes on the Great Barrier Reef (GBR). The LTMP has estimated reef fish density using UVS since the early 1990s (Halford & Thompson, 1994). However, apart from some species of commercial value (e.g. coral trout, *Plectropomus* spp.), the lengths of fishes have not been estimated.

Measures of fish length are useful as they can provide information on trends in biomass and population age structure (MacNeil et al., 2015; Emslie et al., 2015), spawning potential (Carter et al., 2014), sex ratio (Carter et al., 2017) and fishing pressure (Williamson, Russ & Ayling, 2004; Graham et al., 2007; Russ et al., 2008; Emslie et al., 2015). Importantly, estimates of biomass of fishes grouped by their ecological role (e.g. grazing by herbivorous fishes—Hoey & Bellwood, 2008), inform approaches to management based on ecological function (McClanahan et al., 2011; MacNeil et al., 2015). However, it is not known whether altering existing UVS protocols to include estimating fish lengths affects counts. For example, adding estimates of the lengths of each individual fish to UVS will likely alter an observer’s search pattern as handling times for recording individual fish will have increased, particularly when schooling fish are abundant. The added task of estimating each fish’s length could result in generally altered fish counts. The observer’s swimming speed is also known to influence fish counts due to either the efficiency of the observer or the presence of the diver (Thresher & Gunn, 1986; Fowler, 1987; Lincoln Smith, 1988). Therefore, if estimates of fish length are to be incorporated into existing LTMPs, it is important to ensure that this methodological change does not alter counts, which if it did, would necessitate the calculation of a conversion factor in order to make valid comparisons with historical data that did not estimate fish lengths.

Here we assess the magnitude and duration of the effect of the presence of divers on estimates of fish density and diversity in UVS and examine whether incorporating length estimations alters those estimates. Specifically, our objectives were:

1. To quantify changes in estimates of abundance and species richness of reef fish assemblages due to the presence of divers and to model the time taken for reef fish communities to return to levels recorded before diver disturbance.
2. To determine whether there are any differences in estimates from visual surveys of reef fish abundance alone compared with surveys where the lengths of fishes were estimated as well.

## Methods

### Ethics statement

The AIMS provided full approval for this purely observational research. This research was conducted under Permit Number G12/34872.1 issued by the GBR Marine Park Authority.

### Field surveys

**Figure 1:**
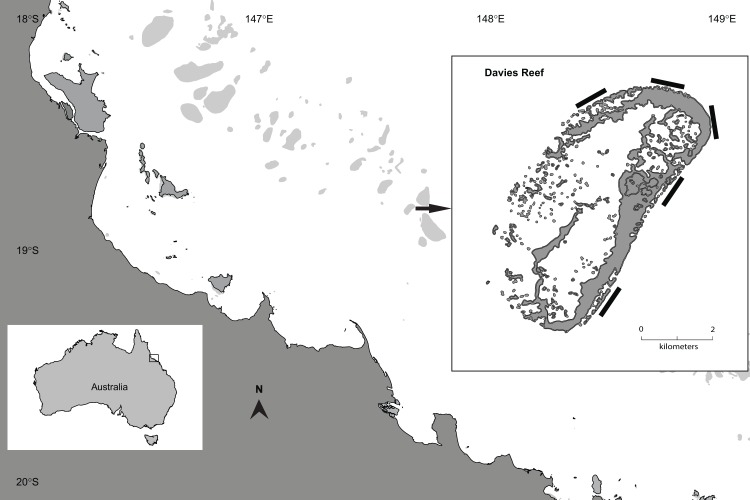
Study location. Map showing the location of the study sites.

This study took place in August 2016 at Davies Reef (18°49′S, 147°38′E), a mid-shelf platform reef in the central GBR (Fig. 1). This reef is designated as a Conservation Zone within the GBR Marine Park, where limited line and spearfishing are allowed. Underwater visibility is typical of mid-shelf reefs in the central GBR (10–15 m), and at 100 km from the coast is visited irregularly by tourism operators and occasional spear and line fishers when weather conditions permit. Ten 50 × 5 m transects located 6–9 m deep on the reef slope, were surveyed at each of five sites around the reef perimeter between 8 am and 4 pm. The minimum distance between each transect was 10 m. At all sites except the first, surveys consisted of an ‘outward leg’ where the lead diver recorded fish data and a second diver followed behind deploying the transect tape. Upon completion of the 10 transects, the divers ascended to the boat. Following a surface interval consistent with dive procedures, which produced variable intervals between surveys (Fig. S1), the same divers re-entered the water and conducted the ‘return leg’ where they re-surveyed the same 10 transects in reverse order with the lead diver again surveying the fishes while the second diver retrieved the transect tapes. The lead diver recorded the abundances of individual coral reef fish species from eight families using one of two instantaneous techniques (counting with or without estimating total length of the fishes). The technique and lead diver were alternated after each transect. At the first site on the first day, an outward leg was swum, but the return leg was precluded by adverse weather. The complete surveys (outward and return legs) were conducted the next day, thus data from site 1 was modelled using all 30 transects. The study was conducted by three highly trained observers, each with greater than 3,000 h experience in conducting UVS of reef fishes. All three observers were alternated so that they all completed the same number of transects over the course of the study.

### Analysis of differences between methods, effects of diver presence and observer comparison

To estimate the effects of initial diver presence (*TPR*), survey method (lengths taken/not-taken; *MET*), survey direction (*LEG*), and observer (*OBS*) on estimates of abundances of specific fish groups, we developed a Bayesian hierarchical model of the abundances of fish groups in repeated surveys of the transects in each site. The fish groups were Acanthuridae, Chaetodontidae, Labridae, Lethrinidae, Lutjanidae, Scarinae, Serranidae, Siganidae and Zanclidae. We also included total richness and total abundance of fishes from all groups per transect. Lethrinids were excluded from analyses due to their extremely low numbers and Scarinae were analysed separately from other Labridae, the family in which they currently are placed taxonomically (Westneat & Alfaro, 2005; Choat et al., 2012), as they are functionally distinct. For each of the fish groups, we modelled counts for each survey (*k*) of each transect (*j*), within site (*i*), using a negative binomial distribution, written in Bayesian notation as:

$$\eqalign{ & {Y_{ij}}\sim{\rm{NB}}\left( {{{\rm{\lambda }}_{ij}},{\rm{\alpha }}} \right) \cr & {{\rm{\lambda }}_{ijk}} = {{\rm{e}}^{{{\rm{\beta }}_{0ij}} + {{\rm{\beta }}_1}MET + {{\rm{\beta }}_2}TPR + {{\rm{\beta }}_3}OBS + {{\rm{\beta }}_4}LEG}} \cr & {{\rm{\beta }}_{0ij}}\sim{\rm{N}}\left( {{{\rm{\eta }}_{0i}},{{\rm{\sigma }}_j}} \right) \cr & {{\rm{\eta }}_{0i}}\sim{\rm{N}}\left( {{{\rm{\gamma }}_0},{{\rm{\sigma }}_i}} \right) \cr & {{\rm{\beta }}_{1,2,3,4}},{{\rm{\gamma }}_0}\sim{\rm{N}}\left( {0,100} \right) \cr & {{\rm{\sigma }}_j},{{\rm{\sigma }}_i}\sim{\rm{U}}\left( {0,100} \right) \cr & {\rm{\alpha }}\sim\Gamma \left( {0.001,0.001} \right) \cr}$$

This included a common shape parameter (α) among transects. We ran this model for each of the fish groups in succession. Parameter traces were stored for subsequent inference. We examined each set of traces and used the criterion that Geweke scores from multiple chains were close to one (Geweke, 1992) to examine model convergence. Models were run for 20,000 iterations using the Metropolis sampler in PyMC3 (Salvatier, Wiecki & Fonnesbeck, 2016). Inferences were based on posterior medians of the parameter effect sizes, along with 50% and 95% uncertainty intervals (UIs). Effect sizes are the per-unit increase in each response, given a log-link function within a negative binomial model, and are on a natural log scale. They were calculated as the difference between parameter estimates associated with (1) methods (counts with and without length estimates), (2) diver disturbance (outward and return legs) and (3) observers, sampled from the posterior distribution of the model. No effect was inferred if UIs intersected zero.

### Density dependent effects on differences between methods

We examined whether total fish abundance influenced the accuracy of counts when fish lengths were and were not estimated. First we calculated the difference in total fish abundance between sequential transects within each site in which fish lengths were and were not estimated. We then standardised the difference by expressing it as a percentage of the sum of the two transects. We then used the standardised difference as the response variable in a Bayesian generalised linear mixed model (glmm) with total abundance as the fixed factor, and survey direction (outward/return leg), site and transect as random factors. We modelled the response against a Gaussian distribution for 1,500 iterations for each of three chains after a warm-up of 500 iterations and a thinning interval of 2 using the Bayesian regression models using STAN (brms) package in R 3.3.3 (R Core Team, 2017). Inferences were based upon the mean slope of the predictor variable and associated UIs, and no effect was inferred if UIs intersected zero.

### Return to pre-disturbance levels

To estimate the time required for the abundance of fishes to return to pre-disturbance levels, we calculated how long it would take for a second survey to give an estimate equal to the abundance recorded in the initial one, based on the posterior medians for the direction and time-lag parameters from our model (i.e. assuming no observer or method effects). With the exception of the Labridae, this calculation involved projecting return times beyond the longest observed interval between surveys (∼4 h). When making such projections, we assumed that the relationship between estimates of abundance and inter-survey interval remained constant.

## Results

**Figure 2:**
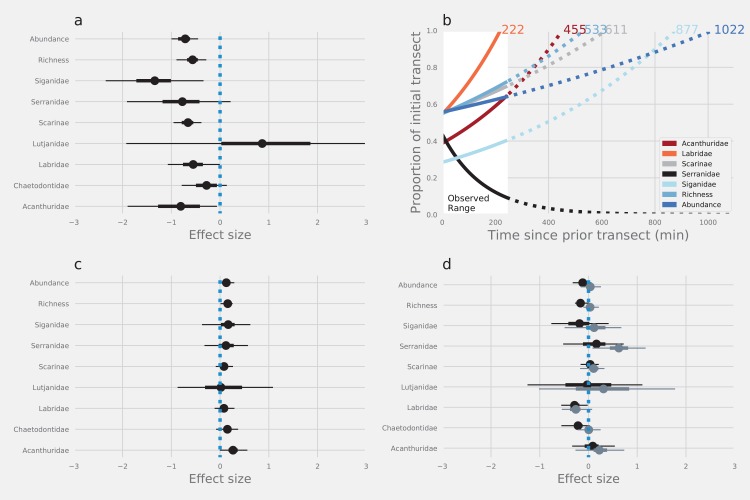
The effects of diver disturbance on estimates of reef fish abundance and diversity. The effects on estimates of reef fish abundance and species richness due to (A) diver disturbance: difference between abundance in initial and subsequent surveys (C) method: counts with and without estimation of lengths, and (D) observer: differences between Observer 1 and 2 (black points) and Observer 1 and 3 (grey points). Points are modelled median log effect sizes with associated 50% (thick error bars) and 95% (thin error bars) Uncertainty Intervals (UIs). Statistical inferences of difference depend on whether UI intersect zero or not. Data were modelled using Bayesian hierarchical linear mixed models and effect sizes are the per-unit increase in each response, given a log-link function within a negative binomial model, and are on a natural log scale. Panel (B) displays the modelled time for abundance of different groups of reef fishes to return to initial levels (outward leg).

The first pass of SCUBA divers along transects led to a substantial reduction in the abundance and number of species of reef fishes that were recorded in subsequent resurveys. Estimates of total abundance, species richness and the abundance of four of seven families were reduced by between 24% and 73% on the return leg compared with estimates from the outward leg (Fig. 2A; Table 1). The herbivorous rabbitfishes (Siganidae), surgeonfishes (Acanthuridae) and parrotfishes (Scarinae), and the predatory wrasses (Labridae) were affected most. Although the abundance of butterflyfish (Chaetodontidae) and fishery target species like groupers (Serranidae) was reduced on the return leg, UIs around the medians intersected zero, providing much weaker evidence of any diver effect on these fishes. Similarly, there was substantial overlap of UIs and zero providing strong evidence that there was no effect of diver disturbance on the snappers (Lutjanidae—Fig. 2A; Table 1).

**Table 1:** Estimates of abundance and species richness of reef fishes from UVS on transects at Davies Reef.

Taxa	Direction	Method	Mean	S.E.	Lower	Upper
Total abundance	Outward	Count	44.24	3.37	40.87	47.61
Total abundance	Outward	Length	48.36	5.16	43.2	53.52
Total abundance	Return	Count	31.72	3.94	27.78	35.66
Total abundance	Return	Length	22.68	1.94	20.74	24.62
Species richness	Outward	Count	15.44	0.7	14.74	16.14
Species richness	Outward	Length	16.64	0.88	15.76	17.52
Species richness	Return	Count	11.72	0.75	10.97	12.47
Species richness	Return	Length	10.64	0.52	10.12	11.16
Acanthuridae	Outward	Count	9.25	1.52	7.73	10.77
Acanthuridae	Outward	Length	10.04	2.36	7.68	12.4
Acanthuridae	Return	Count	5.83	1.42	4.4	7.25
Acanthuridae	Return	Length	4.48	1.08	3.39	5.56
Chaetodontidae	Outward	Count	5.27	0.56	4.71	5.83
Chaetodontidae	Outward	Length	4.36	0.5	3.86	4.86
Chaetodontidae	Return	Count	4.5	0.54	3.96	5.04
Chaetodontidae	Return	Length	3.71	0.48	3.23	4.19
Labridae	Outward	Count	3.33	0.47	2.86	3.8
Labridae	Outward	Length	2.88	0.37	2.5	3.25
Labridae	Return	Count	2.59	0.39	2.2	2.98
Labridae	Return	Length	2.32	0.34	1.98	2.66
Lethrinidae	Outward	Count	1.2	0.2	1.0	1.4
Lethrinidae	Outward	Length	1.0	0.0	1.0	1.0
Lethrinidae	Return	Count	2.0	–	–	–
Lethrinidae	Return	Length	–	–	–	–
Lutjanidae	Outward	Count	4.57	2.06	2.51	6.63
Lutjanidae	Outward	Length	3	1.44	1.56	4.44
Lutjanidae	Return	Count	1	0	1	1
Lutjanidae	Return	Length	2	0.58	1.42	2.58
Serranidae	Outward	Count	1.72	0.28	1.44	2
Serranidae	Outward	Length	2.64	0.43	2.22	3.07
Serranidae	Return	Count	1.12	0.12	1	1.25
Serranidae	Return	Length	1.33	0.21	1.12	1.54
Scarinae	Outward	Count	21.84	2.75	19.09	24.59
Scarinae	Outward	Length	23.16	2.7	20.46	25.86
Scarinae	Return	Count	17.24	2.57	14.67	19.81
Scarinae	Return	Length	11.56	1.26	10.3	12.82
Siganidae	Outward	Count	3.35	0.39	2.96	3.74
Siganidae	Outward	Length	7.67	3.46	4.2	11.13
Siganidae	Return	Count	3.69	1.2	2.49	4.89
Siganidae	Return	Length	2.18	0.44	1.74	2.63

Models indicated that fishes with a strong negative response to divers would take between 222 and 1,022 min to return to pre-disturbance levels of abundance (Fig. 2B), although there was some variation around the modelled estimates (Fig. S2). Counts of fishes that were less affected by divers tended to return to pre-disturbance levels relatively quickly (e.g. Labridae—222 min). Estimates of total abundance took the longest time to return to pre-disturbance levels (1,022 min). Interestingly, we found no evidence that Serranidae returned to the transects within the observation period (245 min), leading to a projection of continued decline from the model (Fig. 2B).

**Figure 3:**
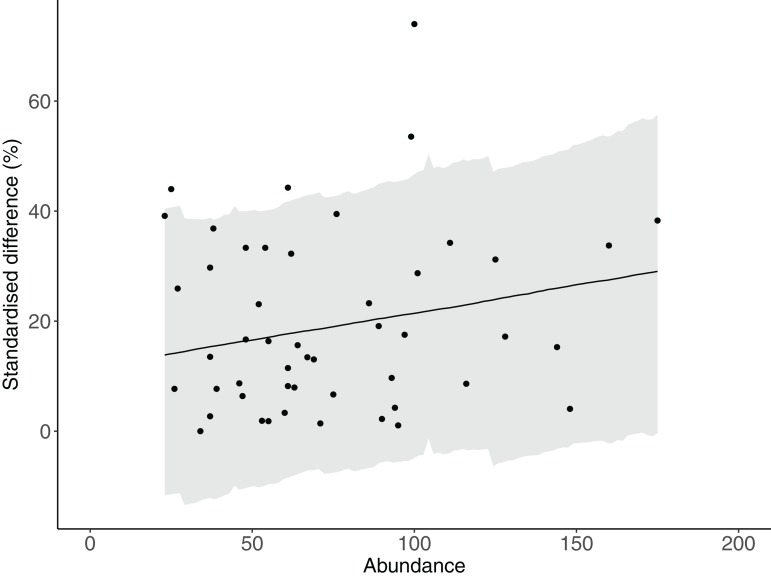
The relationship between total abundance and the difference between estimates of abundance produced by counts with and without estimates of fish lengths.

There was strong evidence that simple counts (without length estimations) of Chaetodontidae, Labridae, Scarinae, Serranidae and Siganidae were unaffected when length estimations were made at the same time. There was weak evidence that estimates of Acanthuridae abundance, total abundance and species richness were lower with the addition of length estimations (Fig. 2C). However, all UIs overlapped zero (Fig. 2C) suggesting there were few statistical differences in estimates of the abundance of reef fishes produced by either method. Additionally, there was no evidence of any density dependent effects on differences in counts between methods (slope = 0.10, lower UI = −0.04, upper UI = 0.25; Fig. 3). There was also little evidence for systematic bias in the estimates of reef fish family abundance among observers in this study (Fig. 2D). There were few instances of difference in abundance and species richness estimates among observers and where this did occur it tended to be for fishes of low abundance such as Labridae and Serranidae (Fig. 2D).

## Discussion

The clear declines in total abundance and species richness of fishes on the return leg of surveys support previous findings of inherent diver-disturbance effects on coral reef fishes during UVS on SCUBA (Chapman et al., 1974; Kulbicki, 1998; Kulbicki & Sarramégna, 1999; Dearden, Theberge & Yasué, 2010). Furthermore, not only were there substantial declines in fish abundance and species richness, but our modelling suggests that these effects maybe much stronger and have far longer durations than previously suspected. Few studies have estimated the expected time for reef fish abundance to return to pre-disturbance levels following diver disturbance during UVS, and to the best of our knowledge, none that have done so for instantaneous counts on belt transects. Contrary to the quick return of fishes (<20 min) following diver disturbance estimated by Dearden, Theberge & Yasué (2010) and also implied by the results of Somerton, Williams & Campbell (2017), our modelling suggested that reef fishes at Davies Reef took between 222 min for the Labridae to 1,022 min for the total assemblage to return to pre-disturbance levels. The discrepancy in our results and those obtained previously may reflected disparities in methodology. Previous studies used video footage (Dearden, Theberge & Yasué, 2010) or repeated counts on transects (Dickens et al., 2011), that were less than 20 min apart. Here we used variable intervals between repeated counts up to 246 min, allowing a more rigorous appraisal of the time taken for fishes to return to pre-disturbance levels of abundance. Interestingly, we found that there was no evidence of return to pre-disturbance abundance of Serranidae during the observation period, suggesting that once this important group of fishery targeted species are disturbed by divers, then any counts of their abundance subsequent to the disturbance will may be underestimated. The return times modelled here indicated that Serranidae never returned to a location once they were disturbed. However this is clearly unlikely to be a true representation, but it does suggest that of all the fishes surveyed here, Serranidae may be particularly sensitive to diver disturbance. Such results suggest for any monitoring program involving estimates of fish abundance, care must be taken to avoid disturbance of fishes prior surveys being undertaken, which will likely result in underestimates of fish abundance.

It is noteworthy that the four fish families that contributed most to total abundance declines have also been reported to decline by 30–70% after diver disturbance on the GBR (Dickens et al., 2011) and contained some species that have been categorised as ‘shy’ and are wary of divers (Kulbicki, 1998). Indeed, negative diver effects have previously been reported (Fowler, 1987; Kulbicki, 1998; Dearden, Theberge & Yasué, 2010; Dickens et al., 2011). These effects imply that UVS may not be the best survey method for shy species and when dealing with these species, observers should utilise other appropriate methods if possible (e.g. remote video), or at least, minimise pre-survey disturbance. Additionally, the laying of transect tapes before surveys commence should be avoided (Dickens et al., 2011). Several taxa showed much weaker effects of diver disturbance. Among these were the butterflyfishes which have been classified previously as diver neutral (Kulbicki, 1998). Our results are in agreement with only very weak evidence of a reduction in their abundance following diver disturbance. In general, results from studies that aim to decipher how observation affects counts of reef fishes will vary according to the methods used, especially as different methods affect a diver’s ability to detect cryptic fishes or shy taxa that have moved away from the diver.

Previous studies have found negative effects of divers (e.g. hiding or flight responses) during UVS of reef fishes (Kulbicki, 1998; Dearden, Theberge & Yasué, 2010; Dickens et al., 2011). Some species may detect and avoid divers before they come within visual range of the fish, so the abundance of such species will be underestimated. The physical presence of divers or surveyor’s tapes are commonly cited as a cause, and in this study, a yellow fibreglass tape was laid down on the outward leg, and not retrieved until the return leg. It is possible that this this persistent physical alteration to the reef could be part of the reason why such long lasting effects were observed in this study. However, negative effects may also be a response to sound of SCUBA exhaust bubbles (Cole et al., 2007). Bubbles are relatively noisy (Lobel, 2001) and their sound is transmitted for long distances in the ocean. The sound of exhaust bubbles occurs within frequency range in which fish hearing is most sensitive (Radford et al., 2005). If fishes associate the sound of bubbles with the presence of divers, this may produce biases in surveys using SCUBA (Lindfield et al., 2014). Both sound and the physical presence of divers probably contribute to the negative response observed in this study, but we are unable to assess their relative importance.

The diver effect may be more pronounced in areas open to fishing, particularly where spear-fishing occurs regularly (Cole, 1994; Kulbicki, 1998). Davies Reef, where our study was conducted, is open to both line fishing and spearfishing, and this may have contributed to the large diver effects recorded in this study, particularly in highly prized species such as the Serranidae. However, the level of spearfishing activity at this reef is unknown, and its contribution to the diver effect we observed could not be assessed. Future studies that compare counts from areas both open and closed to spearfishing will be useful in disentangling the role of diver disturbances.

Encouragingly, this study found very small or no difference in abundance estimates from UVS that did and did not also estimate fish lengths. Changing the methodology of a long running program like the LTMP to include estimates of fish length is desirable to reflect the latest best practices in the majority of contemporary surveys from around the world, many of which contribute to global estimates of fish biomass (Graham et al., 2007; MacNeil et al., 2015). Fish length estimates allows a more in-depth examination of different aspects of reef fish assemblages than is possible using estimates of abundance alone. For example, estimates of fish lengths allow insights into population demographics such as changes in the size structure of populations in response to fishing pressure. Similarly, such data can provide information on the sex ratios, spawning potential and recruitment strength, which are particularly relevant to determining whether populations exploited by fishing are receiving adequate levels of replenishment to avoid overfishing. The conversion of fish lengths to biomass, when coupled with other ecologically relevant information also enables ecologists to quantify important ecological processes (e.g. bite rates plus biomass can estimate rates of herbivory), which can then feed into function based management approaches, which lie at the heart of managing for reef resilience. In this context, it is important that the change of methodology of a long running program like the LTMP provides not only the most useful information it can provide, but also allows valid comparisons of historical data of fish abundance, collected before the change in methodology. Our results also suggest that other long term programs monitoring fish populations that are considering altering their survey protocols to include estimates of fish length should be confident that such changes will not unduly confound their estimates of fish abundance in comparison to historical samples.

## Conclusion

This study has identified significant diver disturbance effects on the estimation of the abundance and species richness of reef fishes using UVS. The abundance of the majority of fish taxa was greatly diminished on the return leg of the surveys and the time taken for abundance to return to pre-disturbance levels was variable among taxa, but in most cases was considerably longer than 3 h and in one case (Serranidae) modelling suggested that there was no return to pre-disturbance levels of abundance within the observation period. This study demonstrates that the effects of divers are real, large and can potentially result in large underestimates in abundance if care is not taken to mitigate such effects. Importantly, we found no or very small differences in abundance estimates derived from UVS that did and did not estimate fish lengths, enabling valid comparisons between monitoring programs that utilise these different methods. For the LTMP which changed methodology after 25 years, it also means comparisons of abundance before and after the change in method are also valid.

## Supplemental Information

10.7717/peerj.4886/supp-1 Supplemental Information 1 Supplemental material.

10.7717/peerj.4886/supp-2 Supplemental Information 2 UVS fish data Davies Reef. Raw data obtained during underwater visual surveys of reef fishes at Davies Reef, Central GBR.

## Additional Information and Declarations

### Field Study Permissions

The following information was supplied relating to field study approvals (i.e. approving body and any reference numbers): This research was conducted under Permit Number G12/34872.1 issued by the Great Barrier Reef Marine Park Authority.

### Data Availability

The following information was supplied regarding data availability: GitHub: https://gist.github.com/mamacneil/f91942c3908890f3982718b1d2d7d249; https://gist.github.com/mamacneil/cc69bae2c1fe08a0915c7fef9a99f950.
