# Supplementary material for: Reef fish communities are spooked by scuba surveys and may take hours to recover
Source: PeerJ. 2018 May 24;6:e4886. doi: 10.7717/peerj.4886 (PMC5971101; doi:10.7717/peerj.4886)
Supplement: Supplemental Information 1 [file peerj-06-4886-s001.docx]

**Supplemental Material**


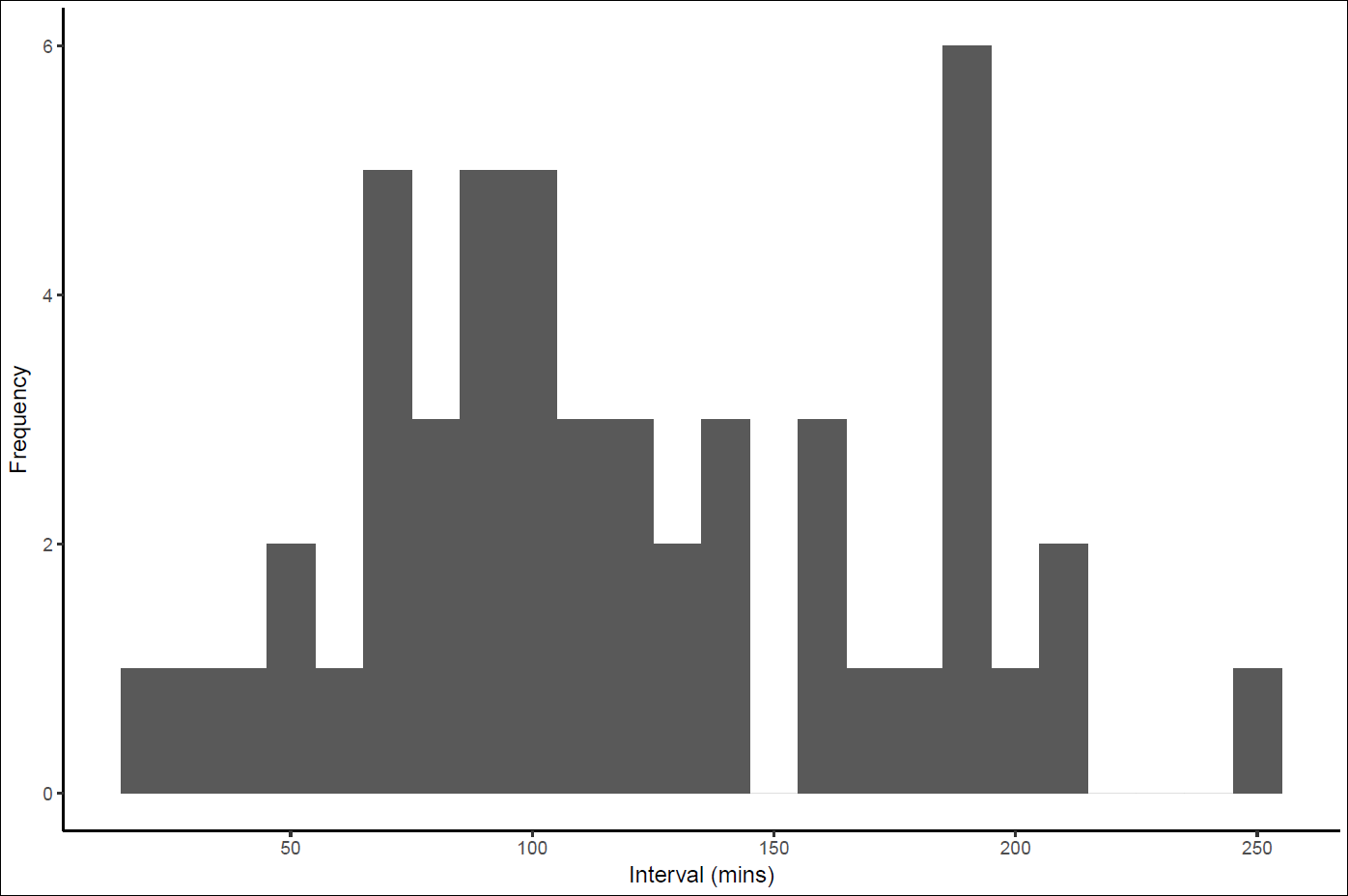


**Supplemental Figure S1** – histogram of interval times between outward and return legs of UVS of reef fishes.


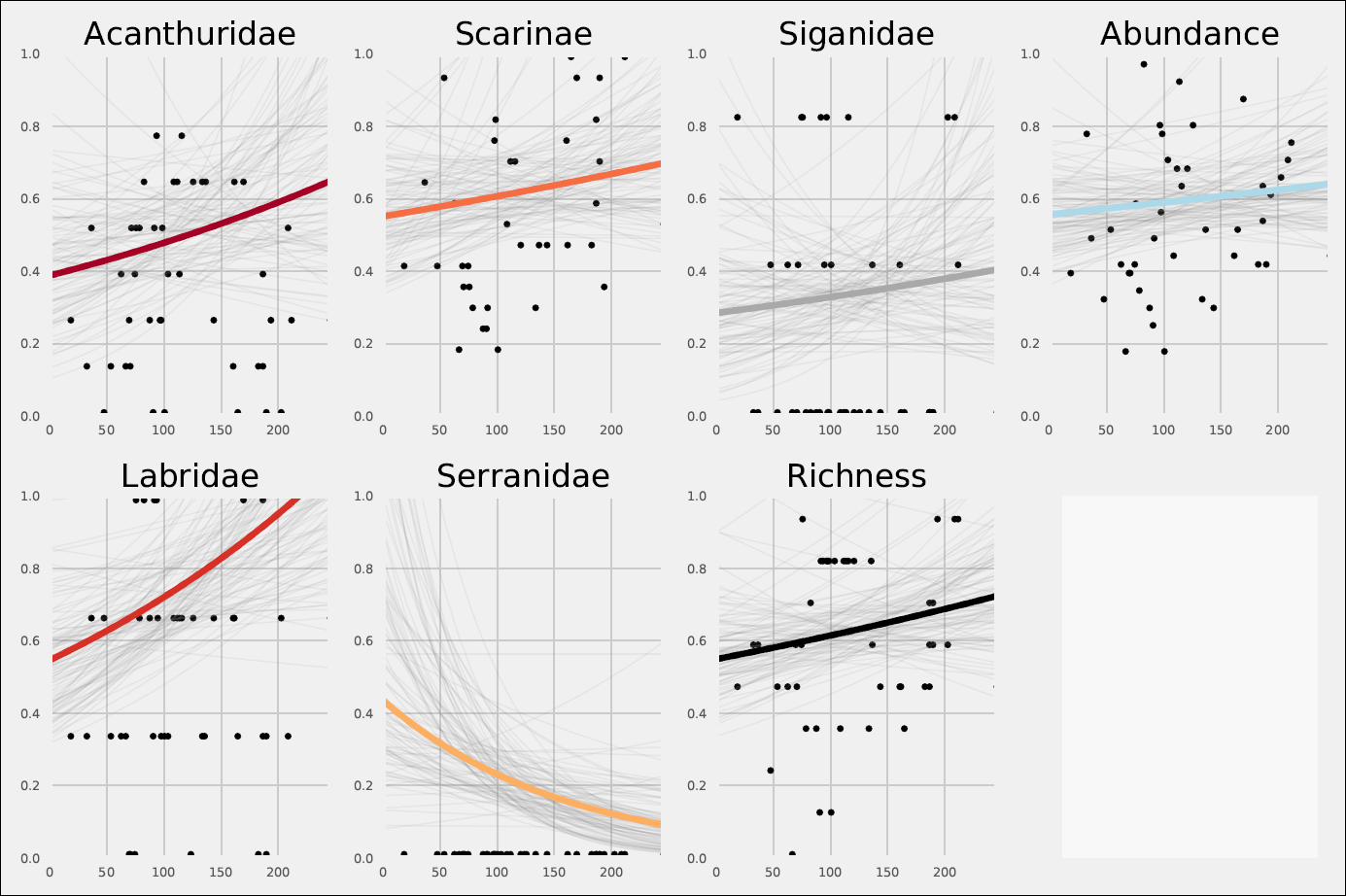


**Supplemental Figure S2** – variability in modelled time taken for fish abundance and species richness to return to pre-disturbance levels. Bold coloured lines are the median response while grey lines are the individual iterations showing the variability in response. Black data points are the estimates of fish abundance on the return leg as a proportion of the initial outward leg.
